# The fiscal value of human lives lost from coronavirus disease (COVID-19) in China

**DOI:** 10.1186/s13104-020-05044-y

**Published:** 2020-04-01

**Authors:** Joses M. Kirigia, Rose Nabi Deborah Karimi Muthuri

**Affiliations:** 1 African Sustainable Development Research Consortium (ASDRC), P.O. Box 6994, Nairobi GPO, 00100 Kenya; 2 Faculty of Health Sciences, University of Pretoria, Pretoria, South Africa

**Keywords:** Coronavirus disease, Fiscal value of human lives, Non-health gross domestic product

## Abstract

**Objective:**

According to the WHO coronavirus disease (COVID-19) situation report 35, as of 24th February 2020, there was a total of 77,262 confirmed COVID-19 cases in China. That included 2595 deaths. The specific objective of this study was to estimate the fiscal value of human lives lost due to COVID-19 in China as of 24th February 2020.

**Results:**

The deaths from COVID-19 had a discounted (at 3%) total fiscal value of Int$ 924,346,795 in China. Out of which, 63.2% was borne by people aged 25–49 years, 27.8% by people aged 50–64 years, and 9.0% by people aged 65 years and above. The average fiscal value per death was Int$ 356,203. Re-estimation of the economic model alternately with 5% and 10 discount rates led to a reduction in the expected total fiscal value by 21.3% and 50.4%, respectively. Furthermore, the re-estimation of the economic model using the world’s highest average life expectancy of 87.1 years (which is that of Japanese females), instead of the national life expectancy of 76.4 years, increased the total fiscal value by Int$ 229,456,430 (24.8%).

## Introduction

China is a member state of the WHO Western Pacific region. It has a population of 1409.29 million and a total gross domestic product (GDP) of Int$ 29,712.83 billion [1].

According to WHO, as at 24 February 2020, there was a total of 79,331 confirmed coronavirus disease (COVID-19) cases in the world, which including 2618 deaths [2]. About 77,262 (97.39%) of those cases and 2595 (99.12%) were in China. Huang et al. [3] study entitled “Clinical features of patients infected with 2019 novel coronavirus in Wuhan, China” revealed that 49% of people who died of COVID-19 were aged 25–49 years, 34% were aged 50–64 years, and 17% were aged 65 years and above.

China’s capacity to contain the spread of COVID-19 hinges on the strength and resilience of its national health system (NHS), disease surveillance system and other systems that address social determinants of health (SDH). The Universal Health Coverage (UHC) service coverage index [4] for China of 76% implies a gap in coverage of essential health services (reproductive, maternal, newborn and child health; infectious diseases; noncommunicable diseases (NCD); and service capacity and access) of 24% [5]. The average of 13 international health regulations (IHR) core capacities (i.e. legislation and financing, coordination and national focal point, zoonotic events, and the human-animal interface, food safety, laboratory, surveillance, human resources, national health emergency framework, health service provision, risk communication, points of entry, chemical events, and radiation emergencies) scores for China is 94%; implying gaps in IHR core capacities of 6% [6]. Approximately, 92% of China’s population uses safely managed drinking water services, implying a gap of 8% [7]. And the population using safely managed sanitation services is 72%, meaning the existence of a coverage gap of 28%. Also, nearly 4.9% of adults (those aged 15 years and above) are not literate [8]. The gaps in NHS (as indicated by coverage of essential health services), disease surveillance (shown in the sub-optimal IHR capacities), and systems that tackle SDH (such as water, sanitation, and education) might hamper China’s efforts expand effective coverage of various preventive interventions against COVID-19.

Therefore, there is a need for economic studies that can be used to contribute towards making a case for investing more resources in the strengthening of NHS, IHR capacities and other systems that tackle SDH. To date, no study has attempted to estimate the fiscal value of human lives lost due to COVID-19. The specific objective of this study was to estimate the fiscal value of human lives lost due to COVID-19 in China as of 24th February 2020.

## Main text

### Methods

#### Analytical framework

This study employed the value of human life analytical framework developed by Weisbrod [9], Landefeld and Seskin [10], Hall and Jones [11], Chisholm et al. [12] and WHO [13]; and applied in the past to estimate the productivity losses associated with Ebola Virus Disease (EVD) in the Democratic Republic of the Congo [14]; deaths associated with non-communicable diseases in Africa [15]; deaths due to neglected tropical diseases in Africa [16]; tuberculosis deaths in Africa [17]; maternal deaths in Africa in 2013 [18]; child mortality in Africa [19]; EVD deaths in West Africa [20]; and maternal deaths in Africa in 2010 [21].

Any individual death from COVID-19 constitutes a permanent loss of potential years of life lost (YLL) to society. According to Murray [22], YLL equals potential limit to life minus the age at death. In the current study, YLL was estimated as the difference between the relevant country’s average life expectancy at birth and age at death from COVID-19.

In line with past studies [14–21], China’s non-health GDP per capita (i.e. the difference between GDP per person and current health expenditure per person) was used as a proxy indicator of the money value of each YLL.

China’s fiscal value of YLL $$\left( {FVYLL_{C} } \right)$$ through COVID-19 deaths is the sum of the potential non-health GDP lost among those aged 25–49 $$\left( {FVYLL_{25 - 49} } \right)$$, those aged 50–64 $$\left( {FVYLL_{50 - 64} } \right)$$, and those aged 65 years and above $$\left( {FVYLL_{65} } \right)$$. Each age group’s FVYLL was obtained by multiplying the total discounted years of life lost, non-health GDP per person in international dollars (Int$) (NGDPC_Int$_) and the total number of coronavirus disease deaths (COVID-19D) for age group [9]. China’s $$FVYLL_{C}$$ associated with COVID-19 deaths was estimated using the eq. 1 and 2 below [14]:

1 $$FVYLL_{C} = \,\,\left( {FVYLL_{25 - 49} \, + FVYLL_{50 - 64} \, + \,FVYLL_{ \ge 65} } \right)\,$$

2 $$\begin{gathered} FVYLL_{j} = \sum\limits_{{t = 1}}^{{T = n}} {\left\{ {\left[ {{1 \mathord{\left/ {\vphantom {1 {\left( {1 + r} \right)^{t} }}} \right. \kern-\nulldelimiterspace} {\left( {1 + r} \right)^{t} }}} \right]} \right.} \times \left[ {NGDPC_{{Int\$ }} } \right] \times \left. {\left[ {COVID - 19D_{j} } \right]} \right\} = \hfill \\ \left\{ {\left[ {{1 \mathord{\left/ {\vphantom {1 {\left( {1 + r} \right)^{1} }}} \right. \kern-\nulldelimiterspace} {\left( {1 + r} \right)^{1} }}} \right]} \right. \times \left[ {NGDPC_{{Int\$ }} } \right] \times \left. {\left[ {COVID - 19D_{j} } \right]} \right\} + \hfill \\ \left\{ {\left[ {{1 \mathord{\left/ {\vphantom {1 {\left( {1 + r} \right)^{2} }}} \right. \kern-\nulldelimiterspace} {\left( {1 + r} \right)^{2} }}} \right]} \right. \times \left[ {NGDPC_{{Int\$ }} } \right] \times \left. {\left[ {COVID - 19D_{j} } \right]} \right\} + \cdots + \hfill \\ \left\{ {\left[ {{1 \mathord{\left/ {\vphantom {1 {\left( {1 + r} \right)^{n} }}} \right. \kern-\nulldelimiterspace} {\left( {1 + r} \right)^{n} }}} \right]} \right. \times \left[ {NGDPC_{{Int\$ }} } \right] \times \left. {\left[ {COVID - 19D_{j} } \right]} \right\} \hfill \\ \end{gathered}$$

where $${1 \mathord{\left/ {\vphantom {1 {\left( {1 + r} \right)^{t} }}} \right. \kern-0pt} {\left( {1 + r} \right)^{t} }}$$ is the discount factor used to convert future non-health GDP losses into today’s dollars; $$r$$ is an interest rate that measures the opportunity cost of lost earnings, which was 3% in the current study [9]; $$\sum\nolimits_{t = 1}^{t = n} {}$$ is the summation from year $$t\, = \, 1$$ to $$t\, = \,n$$; $$t$$ is the first year of life lost, and $$n$$ is the final year of the total number of YLL per COVID-19 death within an age group; $$NGDPC_{\,Int\$ } \,$$ is per capita non-health GDP in Int$ or purchasing power parity (PPP); $$COVID - 19D_{j}$$ is the number of COVID-19 deaths in jth age group, where j = 1 corresponds to the age group 25–49 years, j = 2 to the age group 50–64 years, and j = 3 to the age group 65 years and above in China [9–16]. Future non-health GDP losses were discounted to their present values using 2020 as the base year. China’s mean fiscal value per COVID-19 death was estimated by dividing $$FVYLL_{C}$$ by the total number of COVID-19 deaths borne by the country.

Additional File 1 contains an illustration of how the fiscal value of human lives lost from COVID-19 among age groups 25–49 years, 50–64 years, and 65 years and above were calculated.

### Data and data sources

Data on the number of COVID-19 associated deaths for China (2595) was extracted from the WHO COVID-19 situation report 35 [2]. The life expectancy at birth data for China (76.4 years) was obtained from the WHO world health statistics report 2019 [5]. The GDP per capita data for China (Int$ 21,083.57) was extracted from the IMF World Economic Outlook Database [1]. The current health expenditure (CHE) per capita for China (Int$ 841) data was gotten from the WHO Global Health Expenditure Database [23].

### Sensitivity analysis

As Briggs [24] explains that economic analyses always have some degree of uncertainty, imprecision or methodological controversy. For example: What if discount rates of 5% and 10% had been used, each at a time, instead of 3%? What is the highest life expectancy in the world was used instead of the China average life expectancy? In order to shed light on these two questions, we varied discount rate and life expectancy one at a time to investigate the impact on $FVYLL_{C}$. First, the economic model was alternately re-estimated using 5% and 10% discount rates [14, 25]. Second, the economic model was also re-estimated with the world highest average life expectancy (i.e. the Japanese average female life expectancy) of 87.1 years instead of the national average life expectancy. Thus, the latter was done to gauge the impact of changes in life expectancy on the $FVYLL_{C}$.

## Results

Table 1 shows fiscal value of human lives lost due to COVID-19 in China by 24th February 2020

**Table 1:** Fiscal value of human lives lost due to COVID-19 in China (in 2020 Int$)—assuming different discount rates

Age group in years	Fiscal value of human lives lost at 3% discount rate (Int$)	Fiscal value of human lives lost at 5% discount rate (Int$)	Fiscal value of human lives lost at 10% discount rate (Int$)
25–49	584,440,699	436,046,884	250,013,516
50–64	256,924,436	216,773,094	150,040,667
≥ 65	82,981,659	74,495,627	58,250,604
Total	924,346,795	727,315,605	458,304,787
Average fiscal value per death	356,203	280,275.76	176,611
Average fiscal value per person in population	0.655,895	0.516,087	0.32,520

The 2595 deaths from COVID-19 had a potential total fiscal value of Int$ 924,346,795, i.e. assuming a discount rate of 3% and China’s average life expectancy. Out of which, 63.2% was borne by people aged 25–49 years, 27.8% by people aged 50–64 years, and 9.0% by people aged 65 years and above. The average fiscal value per COVID death was Int$ 356,203 and per person in population was Int$0.000,656.

Re-estimation of the economic model alternately with 5% and 10 discount rates led to a reduction in the expected total fiscal value by Int$ 197,031,189 (21.3%) and Int$ 466,042,007 (50.4%), respectively. This is equivalent to reductions in average fiscal value per death due to COVID-19 of Int$ 75,927and Int$ 179,592.

Table 2 presents a comparison of the fiscal value of human lives lost due to COVID-19 in China assuming the average life expectancy of China and the highest life expectancy in the world.

**Table 2:** A comparison of fiscal value of human lives lost from COVID-19 in China: assuming China’s and world’s highest life expectancies (in 2020 Int$ or PPP)

Age group	Fiscal value of human lives lost at 3% discount rate and assuming the China’s average life expectancy of 76.4 years (Int$)	Fiscal value of human lives lost at 3% discount rate and assuming world’s highest life expectancy of 87.1 years (Int$)
25–49 years	584,440,699	659,302,851
50–64 years	256,924,436	351,570,998
65 years and above	82,981,659	142,929,376
Total	924,346,795	1,153,803,224
Average fiscal value per death	356,203	444,626
Average fiscal value per person in population	0.655895	0.819

Clearly, the re-estimation of the economic model using the highest average life expectancy in the world of 87.1 years, instead of the national life expectancy of 76.4 years, yielded a discounted total fiscal value of Int$ 1153,803,224 and an average fiscal value per death of Int$ 444,626. The use of this higher life expectancy increased the total fiscal value by Int$ 229,456,430 (24.8%).

## Limitations

The study reported in this paper had some limitations. First, the scope of our study was limited to the potential indirect costs associated with premature mortality from COVID-19. It did not include the direct costs, such as cost of diagnosing and treating COVID-19 cases, transport of patients and family members, post-mortem (autopsy), interment, funeral ceremony, etc. Second, our study did not capture the negative macroeconomic (including effects on industry, trade, commerce, tourism/travel, education, investment, consumption, etc.) impact on both the Chinese and the rest of the world economies. Third, according to WHO world statistics report 2019 [5] completeness of cause-of-death primary data for China was 62% in 2017. This implies that the reported number of deaths from COVID-19 might be underestimate; and should that be the case our estimates could be underestimates of the actual fiscal value deaths from COVID-19.

## Supplementary information

**Additional file 1:** Illustration of calculation of fiscal value of human lives lost due to COVID-19 in China.

## Data Availability

All data generated or analysed during this study are included in this published article.

## Abbreviations

CHE: Current health expenditure
COVID-19: Coronavirus disease
COVID-19D_j_: Number of COVID-19 deaths in jth age group
EVD: Ebola Virus Disease
FVYLL: Fiscal value of years of life lost
FVYLL_C_: China’s fiscal value of years of life lost due to COVID-19 deaths
FVYLL_25–49_: Fiscal value of potential years of life lost among those aged 25–49 years
FVYLL_50–64_: Fiscal value of potential years of life lost among those aged 50–64 years
FVYLL_≥65_: Fiscal value of potential years of life lost among those aged 65 years and above
GDP: Gross domestic product
IHR: International health regulations
IMF: International Monetary Fund
Int$: International Dollars or Purchasing Power Parity (PPP)
NCD: Non-communicable disease
NGDPC_Int$_: Non-health GDP per person in purchasing power parity
NHS: National health system
YLL: Potential Years of Life Lost
r: Discount rate
SDH: Social determinants of health
UHC: Universal health coverage
WHO: World Health Organization

## References

[1] International Monetary Fund (IMF). World Economic Outlook Database. IMF, Washington, D.C. 2019. https://www.imf.org/external/pubs/ft/weo/2018/02/weodata/index.aspx. Accessed 4 February 2020.

[2] World Health Organization (WHO). Coronavirus disease (COVID-19) situation report—35. Geneva: WHO; 2020.

[3] Huang C, Wang Y, Li X, Ren L, Zhao J, Hu Y, Zhang L, Fan G, Xu J, Gu X, Cheng Z, Yu T, Xia J, Wei Y, Wu W, Xie X, Yin W, Li H, Liu M, Xiao Y, Gao H, Guo L, Xie J, Wang G, Jiang R, Gao Z, Jin Q, Wang J, Cao B (2020). Clinical features of patients infected with 2019 novel coronavirus in Wuhan, China. Lancet..

[4] WHO and the World Bank (2017). Tracking universal health coverage: 2017 global monitoring report.

[5] WHO (2019). World health statistics overview: monitoring health for the SDGs, sustainable development goals.

[6] WHO (2019). State party annual report.

[7] WHO. Global Health Observatory data repository. Water, sanitation and hygiene. WHO, Geneva. 2020. http://apps.who.int/gho/data/node.main.46?lang=en. Accessed 4 February 2020.

[8] United Nations Development Programme (UNDP) (2018). Human development indices and indicators: 2018 statistical update.

[9] Weisbrod BA (1961). The valuation of human capital. J Political Econ..

[10] Landefeld JS, Seskin EP (1982). The economic value of life: linking theory to practice. Am J Public Health.

[11] Hall RE, Jones CI (2007). The value of life and the rise in health spending. Q J Econ..

[12] Chisholm D, Stanciole AE, Edejer TTT, Evans DB (2010). Economic impact of disease and injury: counting what matters. BMJ.

[13] WHO (2009). WHO guide to identifying the economic consequences of disease and injury.

[14] Kirigia JM, Muthuri RNDK, Muthuri NG (2019). The monetary value of human lives lost through Ebola Virus Disease in the Democratic Republic of Congo in 2019. BMC Public Health..

[15] Kirigia JM, Mwabu GM, M’Imunya JM, Muthuri RDKM, Nkanata LHK, Gitonga EB (2017). Indirect cost of non-communicable diseases deaths in the World Health Organization African Region. Int Arch Med..

[16] Kirigia JM, Mburugu GN (2017). The monetary value of human lives lost due to neglected tropical diseases in Africa. Infect Dis Poverty..

[17] Kirigia JM, Muthuri RDK (2016). Productivity losses associated with tuberculosis deaths in the World Health Organization African Region. Infect Dis Poverty..

[18] Kirigia JM, Mwabu GM, Orem JN, Muthuri RDK (2016). Indirect cost of maternal deaths in the WHO African Region, 2013. Int J Soc Econ..

[19] Kirigia JM, Muthuri RDK, Orem JN, Kirigia DW (2015). Counting the cost of child mortality in the World Health Organization African region. BMC Public Health..

[20] Kirigia JM, Masiye F, Kirigia DW, Akweongo P (2015). Indirect costs associated with deaths from the Ebola virus disease in West Africa. Infect Dis Poverty..

[21] Kirigia JM, Mwabu GM, Orem JN, Muthuri RDK (2014). Indirect cost of maternal deaths in the WHO African Region in 2010. BMC Pregnancy Childb.

[22] Murray CJL (1994). Quantifying the burden of disease: the technical basis for disability-adjusted life years. Bull World Health Organ.

[23] WHO. Global Health Expenditure Database. WHO, Geneva. 2020. http://apps.who.int/nha/database/Select/Indicators/en. Accessed 4 February 2020.

[24] Briggs AH, Drummond MF, McGuire A (2001). Handling uncertainty in economic evaluation. Economic evaluation in health care: merging theory with practice.

[25] Drummond MF, Sculpher MJ, Torrance GW, O’Brien BJ, Stoddart GL (2007). Methods for the economic evaluation of health care programmes.

