# Supplementary material for: The fiscal value of human lives lost from coronavirus disease (COVID-19) in China
Source: BMC Res Notes. 2020 Apr 1;13:198. doi: 10.1186/s13104-020-05044-y (PMC7110291; doi:10.1186/s13104-020-05044-y)
Supplement: Supplementary file 1 — Additional file 1: Illustration of calculation of fiscal value of human lives lost due to COVID-19 in China. [file 13104_2020_5044_MOESM1_ESM.docx]

**Additional File 1: Illustration of calculation of fiscal value of human lives lost due to COVID-19 in China**

The example below illustrates how fiscal value of human lives lost from COVID-19 among age group 25-49 years (FVYLL_24-49_) in China:

1. Total number of COVID-19 deaths among age group 24-49 years as of 24 February 2020 (COVID-19D_25-49_) = 1265.85365853659 (which from 2,595 times 0.487804878)
2. Average age at death among 24-49 years old (AAD_24-49_), i.e. (25+49)/2 = 37 years
3. China’s life expectancy at birth (LE) = 76.4 years
4. China’s per capita GDP (GDPPC) = Int$21,083.57
5. Per capita current health expenditure (CHEPC) = Int$841
6. NGDPC = GDPPC − CHEPC = Int$ 21,083.57 – 841 = Int$ 20,242.57
7. Discount rate ( r ) = 3%
8. Undiscounted years of life lost by a person aged 24-49 years (YLL ) = LE – AAD_24-49_ = 76.4 – 37 = 39.4 years
9. Discounted years of life lost (DISYLL) = 22.80821513
10. FVYLL_24-49_ = DISYLL x NGDPC x COVID-19D_25-49_ = 22.80821513 x 20,242.57 x 1265.85365853659 = Int$ 584,440,699.

We built above formulas in Excel software to obviate inaccuracies. Therefore, fiscal values for the COVID-19 deaths among age groups 50-64 years and 65 years and above were calculated in a similar manner.
